# A Reflective Verbalization Strategy to Trigger Alternative Diagnostic Hypotheses

**DOI:** 10.1111/jep.70267

**Published:** 2025-09-08

**Authors:** Sho Isoda, Taro Shimizu, Tadayuki Hashimoto, Fumio Shimada, Miwa Misawa, Tomio Suzuki

**Affiliations:** ^1^ Department of General Medicine Osaka Medical and Pharmaceutical University Hospital Takatsuki Osaka Japan; ^2^ Psychology Division, Graduate School of Human Science Ritsumeikan University Ibaraki Osaka Japan; ^3^ Japan Diagnostic Excellence Team Japan; ^4^ Department of Diagnostic and Generalist Medicine Dokkyo Medical University Hospital Mibu Tochigi Japan; ^5^ Harvard Medical School Massachusetts USA

**Keywords:** creativity, debiasing, diagnostic excellence, gut feeling, insight, reflective verbalization

## Abstract

**Rationale:**

Physicians sometimes encounter various types of gut feelings (GFs) during clinical diagnosis. The type of GF addressed in this paper refers to the intuitive sense that the generated hypothesis might be incorrect. An appropriate diagnosis cannot be obtained unless these GFs are articulated and inventive solutions are devised. Thus, the method of articulating GFs is critical.

**Aims and Objectives:**

The current study proposes reflective verbalization (RV) to help healthcare professionals capitalize on their GF. In cognitive psychology, RV is the process of verbalizing one's thoughts and feelings through metacognition, to promote deeper understanding and insight problem‐solving. When applied to clinical reasoning, RV can help doctors verbalize their GFs, refining their diagnostic hypotheses.

**Method:**

To address GFs systematically using RV, we introduce the DATES approach, comprising five perspectives: Degree, Abandoned, Time course, Excess, and Shortage. Each perspective prompts physicians to compare their patient's information against typical illness scripts, ensuring no detail is omitted or overlooked.

**Results and Conclusion:**

The tool also aids physicians in considering possible differential diagnoses for one or more of these elements. This guiding tool may aid physicians in overcoming biases, including confirmation and anchoring biases, thus improving diagnostic accuracy. This tool is useful for healthcare professionals who wish to improve their clinical reasoning and decision‐making abilities, particularly when they encounter inexplicable contradictions in their diagnostic hypotheses.

## 1 Introduction

Various studies have explored gut feelings (GFs) in the process of clinical reasoning. When the problem representation of a case differs from the disease image, or the disease gestalt, physicians have GFs [1]. When the problem representation of a case fits the disease gestalt, clinicians feel a sense of reassurance; when they do not fit, they feel a sense of alarm [1]. Some studies have investigated the relationship between GFs and physician management when GFs manifest as a sense of alarm, including the impact of GFs on referral rates to higher‐level medical institutions [1, 2, 3]. However, less attention has been paid to how GFs can be used to generate or refine diagnostic hypotheses. In other words, while GFs have been shown to influence what clinicians do in response to diagnostic uncertainty (i.e., management), it remains unclear how GFs can inform what clinicians think, i.e., their diagnostic reasoning. This lack of exploration may stem from the absence of effective frameworks for articulating the meaning of GFs in diagnostic contexts. It is believed that effective GF articulation could enhance diagnostic accuracy and contribute to diagnostic excellence by fine‐tuning diagnostic hypotheses. In this paper, we propose a strategy to encourage GF verbalization by comparing patient information from different perspectives and utilizing cognitive psychology theories.

### 1.1 Importance of Capitalizing on Gut Feeling

During clinical reasoning—even when formulating diagnostic hypotheses—one may perceive an unexplained inconsistency or feel that something is wrong [1, 2], which is commonly articulated as GFs. Even if one becomes aware of such GFs, if the one is unable to articulate them and devise alternate solutions, their diagnosis may be inaccurate, potentially resulting in failure to achieve diagnostic excellence. Herein, we adopted the following definition for GFs: unverbalized information that does not meet the diagnostic hypothesis [1, 2]. However, due to confirmation and anchoring biases, physicians occasionally disregard GFs that go against their diagnostic assumptions. For example, in a case recalled by a physician, an original diagnosis of infectious enteritis was made based on the patient's clinical presentation: a 30‐year‐old man attending the clinic with acute fever, vomiting, abdominal pain, profuse diarrhea, and tachycardia. The physician felt uncomfortable with his initial decision, experiencing a GF during examination that something was wrong. Despite the GF, the physician determined that infectious enteritis remained highly likely, because of his inability to interpret his GF precisely. The physician discharged the patient after providing symptomatic treatment. However, the patient returned to the emergency room after losing consciousness and was ultimately diagnosed with toxic shock syndrome. Upon reflection, the reasons for the GF experienced were verbalized as relative to tachycardia, mildly elevated respiratory rate, and slight generalized body reddening. This vignette highlights the importance of introspection when interpreting GFs and developing a different, more useful diagnostic hypothesis.

Verbalizing GFs before making treatment or management decisions might help overcome confirmation and anchoring biases. A fitting example here is a case where a physician formulated a diagnostic hypothesis of deep vein thrombosis (DVT) based on a patient's symptoms of redness, warmth, swelling, and tenderness in the lower extremities. However, the physician had a GF that this diagnosis might be incorrect. Before making a definitive diagnosis of DVT, the physician realized that the sudden onset of lower leg pain deviated from DVT symptomatology while suggesting a ruptured Baker's cyst. The case further highlights the importance of recognizing and capitalizing on GFs, because they can lead to more useful alternative diagnostic hypotheses. Articulated GFs can liberate individuals from prior assumptions and constraints, allowing physicians to reframe problems and discover solutions. This process of viewing a problem from a new perspective is referred to as creative thinking [4]. When a solution is recalled through such creative thinking, it is sometimes accompanied by a feeling of sudden realization or joy—an “Aha!” experience [4, 5, 6, 7]. Solving a problem with this kind of Aha moment is known as insight problem‐solving [4, 5, 6, 7]. Even if one is able to view things from a new perspective (engage in creative thinking), it is possible that no Aha experience occurs [4].

### 1.2 Reflective Verbalization (RV)

**Figure 1:**
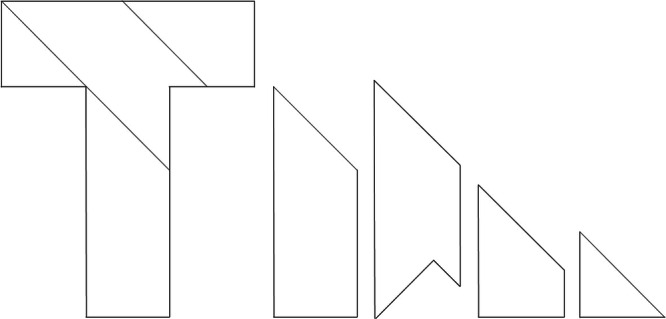
T‐puzzles based on Kiyokawa and Dienes [9]. A T‐shape can be assembled using the four pieces on the right. The solution rate in the reflective verbalization (RV) condition was higher than that in the irrelevant verbalization condition [8].

GFs are often used without being verbalized, remaining as a vague sense of alarm that “something is wrong.” [1] If GFs were explored more analytically and articulated after reflection, they could be exceptional aids in problem‐solving. Reflective verbalization (RV), whereby thoughts and feelings are verbalized through metacognition to gain a deeper understanding of one's experience, is a strategy that can facilitate finding solutions [8]. A T‐puzzle is a common example of RV is explained by a T‐puzzle. The puzzle consists of four pieces (shown on the right in Figure 1) [9], which are combined to form a T‐letter shape (shown on the left in Figure 1) [9]. Kiyokawa and Nagayama used the T‐puzzle in their study, instructing participants to reflect on their previous efforts with it 5 min after beginning [8]. Participants were then asked to write the sentence “this task may not be solved in this way,” which ultimately enabled them to solve the puzzle [8].

Through reflective verbalization that explores alternative solutions, participants can grasp the problem on a meta‐level and from other perspectives, which helps them break free from previously held assumptions and facilitates the generation of new solutions. Thus, RV is a problem‐solving process that involves reflective problem‐solving methods by verbalizing alternative solutions. If GFs are the objects to be verbalized during RV, the diagnostic process can be completed by identifying and verbalizing inconsistencies in the cognitive process through metacognition. Furthermore, to solve the T‐puzzle, problem solvers often need to think from a new perspective and insight, so they are frequently used in cognitive psychology research related to creative thinking and insight. Therefore, verbalizing GFs through RV may support creative thinking and insight, helping to generate novel solutions. Reflection is also important for improving diagnostic accuracy [10]. Reflective practitioners suspend judgment and re‐evaluate their initial hypotheses when they recognize a discrepancy between expected and observed findings [11]. This process involves reframing the problem and generating alternative diagnostic hypotheses. Many studies on reflection have employed clinical scenarios in which all information necessary for an accurate diagnosis is already provided, making them less representative of real‐world practice [11]. In reality, clinicians often encounter cases accompanied by non‐verbalized GFs, and articulating these GFs may serve as a starting point for reflective thinking by complementing incomplete patient information [12]. While many existing approaches aim to support the articulation of clinicians' implicit knowledge, to our knowledge, no previous study has proposed a structured framework that supports the reflective verbalization of gut feelings—defined here as non‐verbalized information that contradicts an initial diagnostic hypothesis—with the specific aim of stimulating the generation of alternative diagnostic hypotheses.

## 2 Proposed Strategy: Application of RV

When patient information differs from the typical illness script of a given disease, a physician may experience a GF [1]. Illness scripts comprise pathophysiology, epidemiology, time course, subjective and objective clinical diagnosis, and management plan [11, 13]. Therefore, the “difference” could stem from one of these components. In this regard, comparing the illness script of a suspected disease with the actual disease is useful in triggering GFs; this process is in fact natural for many physicians. To evaluate the “difference” between the confronting case and the actual illness script, it is necessary to have an analytical view of the ways in which the case differs from the actual illness script. We introduce the following factors to engage in RV in the diagnostic process: Degree, Abandoned, Time course, Excess, and Shortage. We believe that, by using these five RV perspectives, each element of an illness script [8] can be comprehensively compared with corresponding patient information, allowing healthcare professionals' GFs to be articulated. Below are examples of how each of these components has been used to generate useful diagnostic hypotheses.^1^

### 2.1 Degree

This refers to checking the degree to which clinical information (i.e., patient characteristics and symptoms) adequately matches the illness script of the suspected disease.

*Example: A woman in her 30s without underlying medical conditions presented with fever and right‐sided neck pain after a traffic accident. Neck pain started after fever onset. It was attributed to trauma, based on the patient's medical history. However, the physician had a GF that its severity was too extreme for a diagnosis of traumatic neck syndrome. Accordingly, the physician reconsidered the possibility that the neck pain might have been unrelated to the trauma and was instead caused by the fever. This ultimately led to diagnosing a deep neck abscess. Further history clarified that the patient had untreated tooth decay, which was the potential infection source*.

### 2.2 Abandoned

This refers to not overlooking findings that may be considered abnormal per the illness script.

*Example: A man in his 40s presented with a sore throat and fever. During examination, a small abrasion on his left lower extremity was noted, although it was believed to be unrelated to the fever. Based on the acuteness of fever onset, bradycardia, sore throat, mild thrombocytopenia, and elevated liver enzyme levels, an acute viral infection was suspected. However, the clinical phenomena did not align with acute viral infection, spontaneously prompting the physician's GF that something was amiss. This led to reevaluating the information regarding the initially disregarded abrasion, which was suspected to have resulted from a tick bite. Further tests confirmed the diagnosis of scrub typhus (tsutsugamushi disease)*.

### 2.3 Time Course

This refers to verifying that the clinical course is similar to that of the suspected disease.

*Example: A man in his 20s presented with right lower abdominal pain and was diagnosed with appendicitis based on symptoms and findings, including peritoneal signs. He received antibiotic therapy and experienced temporary pain relief. However, 2 months later, he experienced recurrent abdominal pain with similar peritoneal irritation findings. The physician had a GF about the clinical course and explored the patient's medical history further, revealing that the patient had previously suffered from “intractable abdominal pain with peritoneal signs” of the same nature. Thus, the physician revisited the diagnosis for recurrent peritonitis. After considering factors such as age and the symptom commonality, the possibility of familial Mediterranean fever was also explored, and genetic testing returned positive*.

### 2.4 Excess

This refers to confirming that there is no extraneous information compared to the illness script of the suspected disease.

*Example: A 70‐year‐old woman presented with acute high fever, productive cough, and right‐sided chest pain. Lung auscultation revealed decreased breath sounds in the right middle lobe. Although Gram staining was not performed, acute pneumonia caused by Streptococcus pneumoniae was suspected. Penicillin G was initiated. However, the following day, the woman's general condition and respiratory status remained unchanged, and she developed hyponatremia and consciousness disturbance. These “excessive” symptoms raised physician concerns. Patient medical history re‐evaluation with her family revealed that she had visited a hot spring facility a few days before symptom onset. Given the presence of hyponatremia and consciousness disturbance, Legionella pneumophila was considered likely. Subsequent testing confirmed a diagnosis of Legionnaires' disease*.

### 2.5 Shortage

This refers to ensuring that there is no missing information compared to the illness script of the suspected disease.

*Example: A morbidly obese woman in her 60s with a history of colon cancer surgery presented to the hospital with sudden‐onset abdominal pain. Given the acute nature of her symptoms, bowel perforation or adhesive small bowel obstruction was suspected. However, no clear signs of peritoneal irritation were present, and bowel sounds were normal without evidence of obstruction. The physician experienced a GF and considered the differential diagnosis of sudden‐onset abdominal pain; consequently, a bilateral lower‐extremity examination was performed, driven by concerns that there may be an impending abdominal aortic aneurysm rupture. The patient's leg pulses were weak, and both legs were slightly cool to the touch. An emergency abdominal CT scan revealed a ruptured abdominal aortic aneurysm*.

These five elements, expressed with the acronym DATES, were extracted from the authors' past successful experiences. Drawing on tangible instances of successful experiences allows physicians to generate instances that facilitate accurate diagnostic processes [14]. Moreover, in general, diagnostically successful cases are more commonly discussed than unsuccessful ones. Hence, the inclusion of not only failure but also successful instances as subjects for consideration inculcates efficiency in assembling cases for strategy formulation. We recommend that physicians verbalize their GFs in all diagnosis situations following the DATES strategy. This may stimulate verbalization of multiple GFs. Based on the verbalized GFs, physicians may generate further diagnostic hypotheses. Until the GFs are verbalized, it is difficult to determine whether history or examination details are inadequate. Employing RV to verbalize GFs may foster better information and examination completion.

## 3 Discussion

Experienced clinicians have long engaged in reflective practices during clinical reasoning, often verbalizing GFs and sharing insights through informal discussions with peers. The DATES strategy is not intended to replace such practices, but rather to complement and systematize them by offering a structured and reproducible tool for articulating GFs. RV can be used to swiftly verbalize GFs through DATES, leading to the effective use of GFs in the early stages of diagnostic reasoning. A benefit of using DATES is that it can help overcome confirmation and anchoring biases. Even after a diagnostic hypothesis is generated, carefully recognizing and verbalizing GFs using DATES would generate more accurate diagnostic hypotheses. Thus, failed hypotheses due to early closure and ignoring of GFs can be overcome, favoring more accurate diagnoses. Another benefit is the improvement of physician clinical reasoning. For physicians with some experience, DATES may shorten the time required to verbalize GFs and produce an accurate diagnosis, with minimal invasiveness. DATES may help them teach beginners how to improve their clinical reasoning. Beginners encounter difficulties even if they have GFs. While it might not be recommended for beginners to rely solely on GFs for clinical reasoning [15], they should learn to deal with GFs appropriately. Using DATES may reinforce the disease gestalt, refine intuition, and make it easier to have GFs. Furthermore, DATES may be applicable not only for individual diagnostic reflection but also within group‐based reflective practices. In settings such as case conferences, Balint groups, or team debriefings, using the DATES framework to articulate and share gut feelings may help participants identify underlying emotional responses, intuitive reactions, and cognitive biases. Such team‐based reflection has been shown to promote collective metacognition [16].

DATES could also be beneficial for nurses. When nurses have GFs during patient care, verbalizing them using DATES can help them accurately report the patient's condition to physicians, which may lead to earlier recognition of the patient's condition and appropriate intervention. Greater accuracy in reporting patients' situations and improving communication quality can lead to earlier recognition of patients' deteriorating medical conditions, thereby improving medical safety [17].

Additionally, DATES can help with the six fundamental principles of care quality (safe, effective, patient‐centered, timely, efficient, and equitable) in the context of diagnostic excellence [18]. Specifically, this strategy may contribute to realizing the “timely” and “efficient” principles. In addition, GF verbalization may contribute to achieving the “safe,” “timely,” and “efficient” principles if it improves interprofessional communication and leads to early recognition of deteriorating patient conditions.

Despite its educational value, the DATES strategy also has limitations. While DATES facilitates GF verbalization, it does not offer a list of specific diagnostic hypotheses. Therefore, based on insight from DATES, revisiting a diagnostic hypothesis on a zero basis is imperative [19]. DATES requires a certain level of clinical experience and mental bandwidth, which may limit its feasibility in time‐pressured settings. Furthermore, DATES may demand structured training for effective adoption in clinical environments. At present, empirical data supporting its diagnostic impact is limited. Future research should assess its implementation, acceptability, and measurable outcomes in real‐world settings.

## 4 Conclusion

The next step for DATES is to validate the strategy itself. Each of the five outlined perspectives must be re‐examined to confirm DATES' usefulness based on the accumulation of successful experiences of improved diagnostic quality. This process will lead to a more precise description and development of the strategy [15].

## Conflicts of Interest

The authors declare no conflicts of interest.

## Data Availability

Data sharing not applicable to this article as no datasets were generated or analyzed during the current study.

## Endnotes

1 The cases in this article were entirely fabricated and designed purely for explanatory reasons. They do not depict factual individuals or circumstances, and any similarity to real‐life people or incidents is coincidental.
